# PReS-FINAL-2330: Canakinumab treatment in patients with HIDS

**DOI:** 10.1186/1546-0096-11-S2-P320

**Published:** 2013-12-05

**Authors:** J Antón, I Calvo, A Robles, J Yagüe, J Aróstegui, R Viana, S Bhansali, K Abrams

**Affiliations:** 1 H. Sant Joan de Déu, Barcelona, Spain; 2 H. La Fe, Valencia, Spain; 3 H. La Paz, Madrid, Spain; 4 H. Clínic i provincial de Barcelona, Spain; 5 Novartis Farmacéutica, S.A., Barcelona, Spain; 6 Novartis Pharmaceuticals Corp, Hanover, USA

## Introduction

Hyper-IgD and periodic-fever syndrome (HIDS) is a recessively inherited-autoinflammatory condition caused by mevalonate-kinase mutations. It is characterized by early-onset (<1 year of age), 4-6 days-long acute inflammatory episodes that typically recur every 4-6 weeks. The main features during episodes include fever, lymphadenopathies, rash, headache, abdominal pain, diarrhea, and a marked acute phase reaction[1,2]. Previous case reports suggested IL-blockade as a potential therapy. Canakinumab (CAN) is a fully-human, selective anti-IL-1β monoclonal-antibody (MoAb). Preliminary clinical and pharmacokinetics (PK) data of CAN-therapy in active HIDS patients is presented.

## Objectives

Primary objective was to assess if CAN reduces the flare-rate during the treatment period (TP) compared with that from the historical period (HP). One secondary objective was to assess the PK and pharmacodynamics of CAN.

## Methods

This is a 6-month open-label CAN TP and a follow-up period lasting until relapse or up to 6-months max. Patients ≥2 years-old with active HIDS, CRP > 10 mg/L, and ≥3 febrile acute-flares in a 6-month HP were included. All received CAN 4 mg/kg (max. 300 mg) Q6-weeks in TP, with only one dose up-titration to 6 mg/kg (max. 450 mg) if a flare occurred during the first 6-weeks. CAN concentrations were determined by ELISA from samples collected at pre-dose, at pre-specified time points during the first-month, and at flares. Population PK analysis was performed using NONMEM^®^-program.

## Results

Nine patients (6F,3M) with a median age of 17.3 years (5-29 years) were enrolled. The median number of flares/patient reduced from 5(3-12) during the HP to 0(0-2) during the TP. Two patients had a total of 3 flares during the TP and both required dose up-titrated, with no flares afterwards during the TP. Seven out of 9 patients flared during the follow-up period at a median of 110 days (62-196) after the last CAN dose. Population PK analysis showed that serum clearance of CAN and its volume of distribution were dependent on bodyweight. The estimated apparent serum clearance (CL/F) of CAN was 0.20 ± 0.041 L/day and the corresponding volume of distribution (V_ss_/F) was 11.6 L. Following the first dose, the mean (SD) observed C_max _was 30.4 ± 8.13 μg/mL. Apparently weight normalized PK parameters were similar to those observed in other diseases. Adverse events (AE) were reported in eight patients, most were mild (76%) or moderate (18%), and none led to CAN discontinuation. Infections, mostly involving the respiratory tract, were the most common type of AE reported. Two patients reported a serious AE (1 HIDS flare hospitalization, and 1 gastrointestinal infection bleed and separate peritonitis).

## Conclusion

In this small study, CAN decreased the flare-rate substantially. PK of CAN in HIDS patients was as expected for a MoAb and the weight normalized PK parameters were comparable to those observed in other patient populations. The AEs reported were manageable. Further study is needed to better define CAN treatment in HIDS.

## Disclosure of interest

J. Antón: None Declared, I. Calvo: None Declared, A. Robles: None Declared, J. Yagüe: None Declared, J. Aróstegui: None Declared, R. Viana Employee of: Novartis Farmacéutica, S.A., S. Bhansali Employee of: Novartis Pharmaceuticals Corp, USA, K. Abrams Employee of: Novartis Pharmaceuticals Corp, USA.
